# A Novel System for the Launch of Alphavirus RNA Synthesis Reveals a Role for the Imd Pathway in Arthropod Antiviral Response

**DOI:** 10.1371/journal.ppat.1000582

**Published:** 2009-09-18

**Authors:** Vasanthi Avadhanula, Brandon P. Weasner, Gail G. Hardy, Justin P. Kumar, Richard W. Hardy

**Affiliations:** Department of Biology, Indiana University, Bloomington, Indiana, United States of America; University of California Riverside, United States of America

## Abstract

Alphaviruses are RNA viruses transmitted between vertebrate hosts by arthropod vectors, primarily mosquitoes. How arthropods counteract alphaviruses or viruses per se is not very well understood. *Drosophila melanogaster* is a powerful model system for studying innate immunity against bacterial and fungal infections. In this study we report the use of a novel system to analyze replication of Sindbis virus (type species of the alphavirus genus) RNA following expression of a Sindbis virus replicon RNA from the fly genome. We demonstrate deficits in the immune deficiency (Imd) pathway enhance viral replication while mutations in the Toll pathway fail to affect replication. Similar results were observed with intrathoracic injections of whole virus and confirmed in cultured mosquito cells. These findings show that the Imd pathway mediates an antiviral response to Sindbis virus replication. To our knowledge, this is the first demonstration of an antiviral role for the Imd pathway in insects.

## Author Summary

Alphaviruses are arthropod-borne viruses maintained primarily in an endemic cycle between mosquitoes and rodents or birds. Transmission to humans may result in wide ranging symptoms from subclinical to fatal encephalitis. While infection of vertebrates causes disease, infection of mosquitoes results in a life-long, persistent infection. In order to examine arthropod host pathways involved in controlling alphavirus infections, we have employed a novel system for the controlled launch of Sindbis virus RNA replication from the genome of the fruit fly, *Drosophila melanogaster*. We present data showing robust replication of a Sindbis virus RNA following its cell-mediated transcription in flies using the UAS-GAL4 misexpression system. Using this system we have genetically demonstrated that the immune deficiency pathway (Imd) suppresses viral RNA replication as a consequence of the activation of the transcription factor Relish. Additionally, we confirmed the activation of the Relish ortholog as a consequence of Sindbis virus infection of mosquito cells. Our work is the first direct demonstration that the Imd pathway plays a role in arthropod antiviral immunity.

## Introduction

Arboviruses are a large group of RNA viruses that are transmitted between vertebrate hosts by arthropod vectors, primarily mosquitoes. Several arboviruses including members of alphavirus and flavivirus genera are important human pathogens causing severe arthritis, encephalitis, and hemorrhagic fever. Arboviruses are distributed globally, but individual virus species tend to have a focused geographic range. In the recent past, some viruses have expanded globally, and have caused more frequent and larger epidemics. For example, a strain of Chikungunya virus (an alphavirus) endemic to Africa caused an epidemic outbreak in the Indian sub-continent and the Indian Ocean islands leading to more than a million cases of disease and hundreds of death [1],[2]. Similarly West Nile virus (a flavivirus) originally isolated from Uganda has caused about 100,000 cases of neuroinvasive disease and numerous deaths in North and South America [3]. The periodic nature of the infections along with increasing morbidity and mortality in several parts of the world poses a persistent public health risk [4],[5]. Restriction of arbovirus transmission may be accomplished by vector control, vaccination, and/or antiviral treatment. However, currently there are few vaccines and no effective antiviral therapies available, nor are there efficient and safe means of vector control, underscoring the need to understand how arboviruses interact with vertebrate and arthropod hosts.

Alphaviruses form an important group of arboviruses that causes human disease. They are divided into two clinical groups; those that cause serious but primarily non life-threatening illness like rash and arthritis and those that cause fatal encephalitis. The arthritogenic viruses include Sindbis, Chikungunya, and O'nyong-nyong viruses, while the encephalitogenic viruses include Venezuelan, western, and eastern equine encephalitis viruses [2],[4],[6],[7]. Alphaviruses replicate efficiently in both arthropod and vertebrate hosts, however the pattern of infection differs in a host-dependent fashion; in vertebrate cells alphaviruses cause an acute cytolytic infection, whereas in mosquito cells the infection is predominantly persistent and non-cytolytic. This observation strongly suggests that the virus interacts with the host cells in different ways. Most studies of alphavirus pathogenesis and host responses have been performed in mammalian systems and there is a great deal of information available regarding the antiviral response in vertebrates [8],[9]. However, less is known about the antiviral immunity against alphaviruses in arthropods.

Innate immunity plays an important role in limiting microbes in arthropods, through humoral responses (production of effector molecules such as antimicrobial peptides [AMP]), physical barriers, phagocytosis, encapsulation, and melanization [10]. *Drosophila melanogaster* has been used as an excellent model to study innate immune responses against pathogens that infect insects. Immune responses to various bacterial and fungal pathogens have been well characterized in *Drosophila* and primarily consist of the Toll and Imd pathways. The Toll pathway is activated by Gram-positive bacteria and fungi. The pathogen associated microbial patterns (PAMPs) such as lysine type-peptidoglycan are recognized by peptidoglycan receptor proteins (PGRPs) and this binding initiates a serine protease cascade. The cleaved form of the cytokine Spätzle activates transmembrane Toll receptor, which directs the phosphorylation and degradation of Cactus, an IκB-like protein that inhibits the NF-κB like transcription factors Dorsal and Dif. Translocation of these transcription factors to the nucleus causes a rapid increase in expression of multiple AMPs including Drosomycin [10]–[15]. The Imd pathway is stimulated by Gram-negative bacteria. When bacterial PAMP's such as monomeric or polymeric diaminopimelic acid peptidoglycan, bind to the transmembrane PGRP-LC receptor [16], a death domain adaptor protein Imd is recruited. Imd binds to dFadd, another death domain protein which in turn interacts with the apical caspase Dredd [17]–[19]. This caspase then cleaves phosphorylated Relish, a NF-κB-type transcription factor [20]. Relish is phosphorylated by the IKK signaling complex, which is itself thought to be activated by TGF-β activated kinase 1 (Tak1) and its adaptor TAK1-associated binding protein2 (Tab2) [21]–[23]. The cleaved N-terminal domain of Relish then translocates to the nucleus and leads to transcriptional activation of several AMPs including Diptericin [11],[20].

In contrast to the abundant information available for fungal and bacterial infections, less is known about how insects respond to viral infections. Recent studies have pointed to the role of RNA interference (RNAi) in generating antiviral immunity in arthropods [24]–[28]. RNAi, is triggered by the recognition of intracellular long double-stranded RNAs (produced during viral genome replication). The endoribonuclease Dicer-2 processes these into small interfering RNA (siRNA). These siRNA duplexes are then separated by R2D2, and incorporated into the RNA-induced silencing complex (RISC) [29]. The guide strand of siRNA targets the RISC complex to complementary single-stranded RNA, which is then cleaved by the RNaseH like enzyme Argonaute 2 (Ago2) [30]. Flies deficient in Dicer-2, R2D2, or Ago2 exhibit increased sensitivity to infection by Flock house virus (FHV) (*Nodaviridae*), *Drosophila* C Virus (DCV) (*Dicistroviridae*), *Drosophila* X virus (DXV) (*Birnaviridae*), and Sindbis virus (*Togaviridae*, alphavirus) [24],[25],[28].

In addition to RNAi, DCV activates the Jak/STAT pathway in *Drosophila*. Global transcription profiles of flies infected with DCV showed induction of a set of genes distinct from the Toll- and Imd-induced target genes. *vir-1* (virus-induced RNA 1) was strongly induced by DCV and its expression was dependent on Hopscotch, the sole Jak kinase of *Drosophila*. Also, flies deficient in Hopscotch, showed increased viral load and sensitivity to DCV infection [31],[32]. Studies using DXV demonstrated the role of the Toll pathway in antiviral response. Infection with DXV leads to a strong induction of Drosomycin, a marker of the Toll pathway. Also a loss-of-function mutant in Dif (NF-κB component of Toll pathway) and gain-of-function mutant in the Toll receptor were more susceptible to viral challenge and allowed increased viral replication [33]. Even though some of the mechanisms by which *Drosophila* controls viral infections are known, the molecular mechanism by which the Jak/STAT and Toll pathways are triggered or the effector mechanisms that control viral infections through these pathways are not yet understood.

The innate immune responses characterized in mosquitoes have been largely based on what is known in *Drosophila*. The mosquito genome has orthologs to the components of the innate immune machinery of *Drosophila*. Keene et.al. have shown that in *Anopheles gambiae*, *ago2* and *ago3* are required for defense against O'nyong-nyong virus [34] while *ago2*, *r2d2 and dcr2* are required for anti-dengue defense in *Aedes aegypti* [35],[36]. RNAi is also important in defense against SIN; silencing RNAi components in *Ae. aegypti* resulted in transient increases in SIN replication [37]. In addition to RNAi, the Toll pathway is also implicated in antiviral defense in mosquitoes. SIN infection induced the expression of Toll pathway-related *rel1* transcription factor (ortholog of *dif*) and genes involved in the vesicular transport in mid-guts of *Ae. aegypti* [38]. A recent study showed that Toll pathway regulates resistance to dengue virus. Microarray analysis of dengue infected *Ae. aegypti* resulted in up-regulation of Toll pathway associated genes. Activation of the Toll pathway through RNAi-mediated silencing of the negative regulator Cactus reduced dengue virus infection level while repression of the Toll pathway through gene silencing resulted in higher dengue virus infection levels [39].

Although studies have begun to address the antiviral response in insects, much more needs to be known in order limit the spread of alphaviruses and other arboviral infections. In the present study we have taken advantage of the genetic tools available in *Drosophila* to study what host factors effect SIN replication. We generated a transgenic fly line that expresses SIN replicon RNA capable of autonomous replication. Previously, transgenic animals expressing viral genomes have been generated and used to study antiviral responses [24],[40]. In the system we generated primary transcription of the replicon is under the control of the UAS/GAL4 system and hence can be launched in a temporally and spatially specific manner that is dependent on the enhancer/promoter driving GAL4 transcription [41]. We have demonstrated that SIN RNA replication can be launched using this system, providing a powerful tool for the genetic analysis of host genes affecting virus RNA replication. The SIN replicon fly line was crossed to fly lines carrying mutations in the innate immune pathways (Toll, Imd and Jak/STAT) to determine the role these pathways play a role in curtailing SIN replication. SIN replication remained unchanged in flies that were heterozygous for NF-κB orthologs Dif and Dorsal (activated by the Toll pathway) however SIN replication was higher in flies heterozygous for Relish. SIN replication was also enhanced in flies heterozygous for upstream members of the Imd pathway. Furthermore, intrathoracic injections of SIN virus into *relish* ^−/−^ flies showed higher viral loads and enhanced replication in mutant flies compared to wild type. These findings demonstrate that the Imd pathway is involved in antiviral defense against SIN and provide the first direct evidence for the involvement of the Imd pathway in antiviral defense in insects.

## Results

### UAS/GAL4 expression of alphavirus replicon RNA

The UAS/GAL4 system allows for targeted gene expression by selective activation of any cloned gene in a wide variety of tissue- and cell-specific patterns [41]. We utilized this system to introduce RNA analogous to the genome of the alphavirus- SIN into *Drosophila*. Alphavirus genomes can be engineered to express heterologous proteins by substituting the structural protein genes with the heterologous protein gene. This replicon RNA is capable of self-replication but is not able to produce infectious virus particles. The SIN replicon used contains the nonstructural protein genes encoding the viral replicase, the 5′- and 3′-UTRs and a subgenomic promoter that directs expression of green fluorescent protein (GFP). A DNA copy of the SIN replicon genome was cloned behind five UAS enhancer sequences and a minimal heat shock promoter. Transcription from these upstream sequences is activated by the yeast transcriptional activator GAL4 expressed from a specific enhancer/promoter, hence primary transcription of the replicon RNA occurs in temporal and spatial pattern analogous to the gene from which the enhancer/promoter driving GAL4 expression was derived.

**Figure 1:**
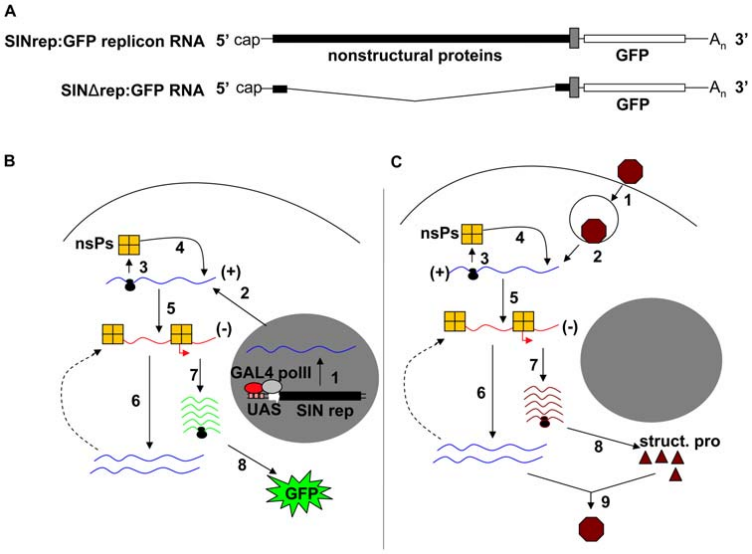
UAS-GAL4 controlled launch of SIN genome replication in *Drosophila*. (A) Schematic of the SINrep:GFP and SINΔrep:GFP constructs used to generate the transgenic flies. SINrep:GFP replicon RNA encodes the 5′cap, the nonstructural proteins the subgenomic promoter, GFP and 3′poly A tail. The SINΔrep:GFP RNA has a large deletion region encoding the nonstructural proteins. The hypothesized launch of alphavirus genome replication under the control of UAS/GAL4 (B) compared to a natural virus infection (C). Introduction of viral genomic plus sense RNA into cytoplasm differs for each system (steps1 and 2). In (B) the viral RNA is introduced after transcription by host RNA pol II where as in infection (C) the RNA is introduced by receptor mediated viral entry and endocytosis. The process of genome replication and subgenomic mRNA expression is the same for each system (steps 3 to 8). In cytoplasm, genomic RNA is translated into nsPs (Step 3). The nsPs copy genomic RNA into a minus-strand RNA (step 4 and 5). The viral replicase complex recognizes the minus-strand RNA and copies it into genomic (step 6) and subgenomic RNA (step 7). The subgenomic RNA encodes for the GFP (B) or structural proteins (C) (step 8). The structural proteins assemble with a copy of the genome into infectious virus particles (C, step 9).

We generated two transgenic fly lines; 1) UAS-SINrep:GFP encodes a SIN replicon RNA capable of GFP expression from the subgenomic mRNA, and 2) UAS-SINΔrep:GFP encodes a mutant form of SIN replicon lacking sequence coding for the nonstructural proteins and hence is incapable of replication. When these fly lines are crossed to “driver” lines expressing GAL4 RNA pol II-mediated transcription of the replicon RNA is activated in the progeny. A schematic of the RNAs encoded by these flies is shown in Figure 1A.

While primary, cell-based, transcription of the SIN replicon RNA is under the control of the UAS/GAL4 system (Figure 1B, steps 1 and 2), GFP expression from this RNA is dependent on the replicon encoded viral RNA synthetic complex comprised of the viral nonstructural proteins. This complex copies the plus-strand replicon RNA (analogous to the SIN genome) into a minus-strand copy which in turn serves as a template for plus-strand RNA synthesis, both full-length replicon RNA and the subgenomic mRNA encoding GFP. Figure 1B shows the hypothesized launch of SIN replicon replication under the control of UAS/GAL4 (Figure 1B) compared to a natural virus infection (Figure 1C). Following the introduction of the viral genomic plus-sense RNA into the cytoplasm, which differs for each system (steps 1 and 2), the process of genome replication and subgenomic mRNA expression is the same for each system (steps 3 to 8), meaning host factors that inhibit or support viral genome replication are the same in each case.

### Cell-based launch of alphavirus RNA replication in *Drosophila*

We crossed UAS-SINrep:GFP line to an Act5C-GAL4 activator line to determine if alphavirus genome replication can be launched by *Drosophila* RNA polymerase II-mediated transcription. Primary transcription of the replicon RNA is dependent on the activity of the Act5C enhancer/promoter to drive expression of GAL4. Act5C was chosen as the driver for these experiments as it has been shown to be broadly expressed during *Drosophila* development and hence provided the greatest opportunity for driving primary transcription of the replicon RNA in a tissue that was permissive for viral RNA replication [42].

**Figure 2:**
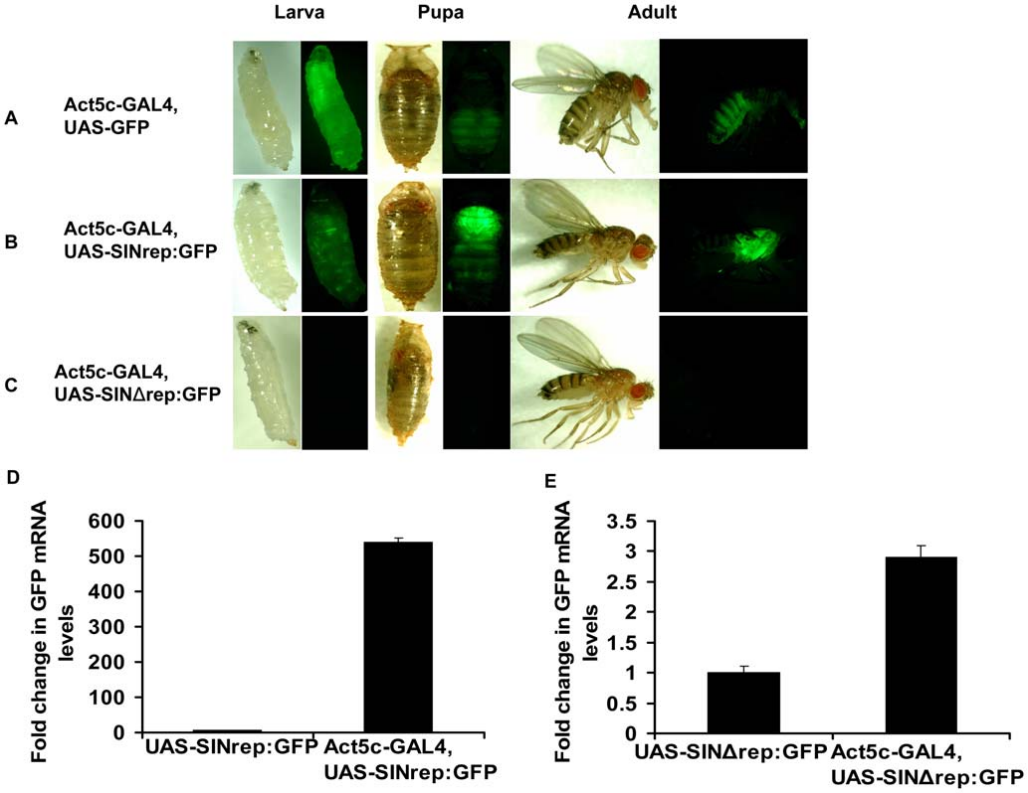
Alphavirus genome replication in *Drosophila*. Bright field and fluorescence images showing pattern of GFP expression in (A) control GFP Act5C-GAL4,UAS-GFP flies, (B) SIN replicon Act5C-GAL4,UAS-SIN:GFP flies and (C) mutant SIN replicon Act5C-GAL4/UAS-SINΔrep:GFP flies during development. (D) Real-time qRT-PCR analysis of GFP mRNA in SIN replicon flies without GAL4 driver (UAS- SINrep:GFP) and with GAL4 driver (Act5C-GAL4,UAS-SIN:GFP) and in (E) mutant SIN replicon flies without (UAS-SINΔrep:GFP) and with GAL4 driver (Act5C-GAL4,UAS-SINΔrep:GFP). The results were normalized to actin, and the value obtained for control flies was considered as one fold. Data shown is representative of three independent experiments. Error bars represent SD.

Expression patterns of GFP in Act5C-GAL4,UAS-SINrep:GFP flies (hereafter referred to as SIN replicon fly) were compared to those in the control Act5C-GAL4,UAS-GFP flies (hereafter referred to as control GFP fly). F1 progeny at various stages of development were examined for GFP expression. In the third instar of control GFP larvae, GFP expression was observed throughout the body with areas of high expression in the anterior end of the larvae, while SIN replicon-derived GFP expression was characterized by punctate areas of high expression throughout the body (Figure 2A and 2B). The expression pattern however changed in late pupae. Act5C driven GFP expression in the control was predominantly in the abdomen with low levels of expression in thorax and head (Figure 2A), whereas replicon derived GFP expression was predominantly in the thorax with little expression in the abdomen and head (Figure 2B). The pattern of expression in adult flies was similar to that of pupae. This result suggests that once viral RNA replication is initiated, the pattern of GFP expression is no longer defined by the pattern of GAL4 expression, and, as long as the cell carries replicon RNA in the cytoplasm and is permissive for viral RNA replication there is no requirement for continuous primary cell-mediated transcription of replicon RNA.

To determine the viral dependence of the GFP expression observed in the SIN replicon expressing flies we crossed the UAS-SINΔrep:GFP line to Act5C-GAL4 line and checked for GFP expression in F1 Act5C-GAL4,UAS-SINΔrep:GFP flies (hereafter referred to as mutant SIN replicon flies). Since the mutant SIN replicon RNA lacks a significant portion of the nonstructural protein coding region, it is not capable of replication and, therefore should be incapable of subgenomic mRNA synthesis and GFP expression. As hypothesized, we observed no GFP expression in the F1 progeny at any developmental stage (Figure 2C). This result demonstrated that the GFP produced in the SIN replicon flies was dependent on the viral non-structural proteins and was therefore a consequence of viral genome replication.

Results of observed GFP fluorescence were confirmed by qRT-PCR of GFP mRNA. Extremely low levels of GFP mRNA were detected in flies containing the DNA copy of the SIN replicon but lacking the GAL4 driver (UAS-SINrep:GFP). In SIN replicon flies with the driver there was a ∼550-fold increase in the level of GFP transcripts (Figure 2D). Additionally replicon derived minus-strand replication intermediates were detected in Act5C-GAL4,UAS-SINrep:GFP by RT-PCR (supplementary data, Figure S1). These data suggest that binding of GAL4 activated primary transcription of the replicon RNA that then replicated autonomously leading to the production of high levels of GFP encoding RNA. In mutant SIN replicon flies without the driver there was again low levels of GFP mRNA detected, however in mutant SIN replicon flies with the driver there was a 2-fold change in GFP transcripts. This change in RNA levels can be attributed to GAL4 activation of primary transcription of mutant replicon RNA (Figure 2E).

*Drosophila* lines expressing replicon RNA, mutant replicon RNA, and GFP were stabilized in order to ensure the co-segregation of the GAL4 and UAS elements. These fly lines were used in the experiments that follow.

### Dicer-2-mediated suppression of alphavirus RNA replication

**Figure 3:**
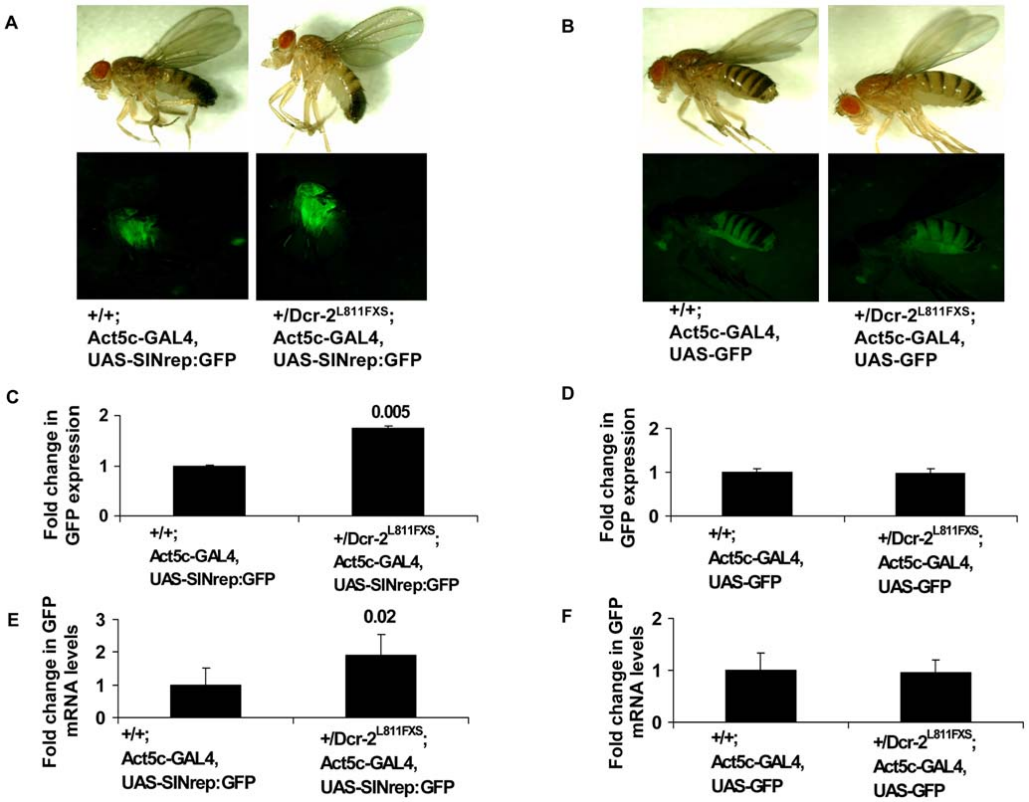
Dicer-2 mutant flies have enhanced alphaviral replication. Bright field and fluorescence images of (A) wild type SIN replicon flies and SIN replicon flies heterozygous for *dcr-2* mutation (*dcr-2^L811FXS^*) and (B) control GFP and GFP flies heterozygous for *dcr-2* mutation. (C and D) GFP expression levels in SIN replicon and control GFP flies measured by fluorometry. Adult flies were homogenized and GFP in homogenates was detected by fluorescence measurements. The value obtained for control flies was considered as one. (E and F) Real-time qRT-PCR analysis of GFP mRNA in SIN replicon and control GFP flies. The results were normalized to actin, and the value obtained for control flies was considered as one. Data shown is representative of three independent experiments. Error bars represent SD.

A previous study showed that flies homozygous for a mutant allele of Dicer-2 (*dcr-2* ^L811FXS^) were more susceptible to SIN infection. Dicer-2 mutant flies when infected with SIN, had increased viral RNA accumulation and higher viral loads compared to wild type flies [24]. To determine if Dicer-2 played a role in controlling the level of SIN RNA replication following host-derived launch we crossed the SIN replicon fly with Dicer-2 mutant flies. The F1 progeny heterozygous for the SIN replicon and Dicer-2 showed increased levels of GFP indicating increased viral replication (Figure 3A). Viral RNA replication was measured by GFP fluorescence and GFP mRNA levels. The fluorescence and mRNA levels were 1.8 and 2- fold higher respectively in flies possessing only one functional copy of Dicer-2 when compared to SIN replicon flies homozygous for wt Dicer-2 (Figure 3C and 3E). There was no change in GFP expression levels or mRNA levels in the control GFP flies heterozygous for Dicer-2 (Figure 3B, 3D and 3F). Our results verified the previously reported role of Dicer-2 and RNAi in controlling SIN infection and confirmed that the UAS/GAL4 system for replicon launch could be used to genetically examine antiviral responses in *Drosophila*.

### Role of antimicrobial pathways in alphavirus replication

**Figure 4:**
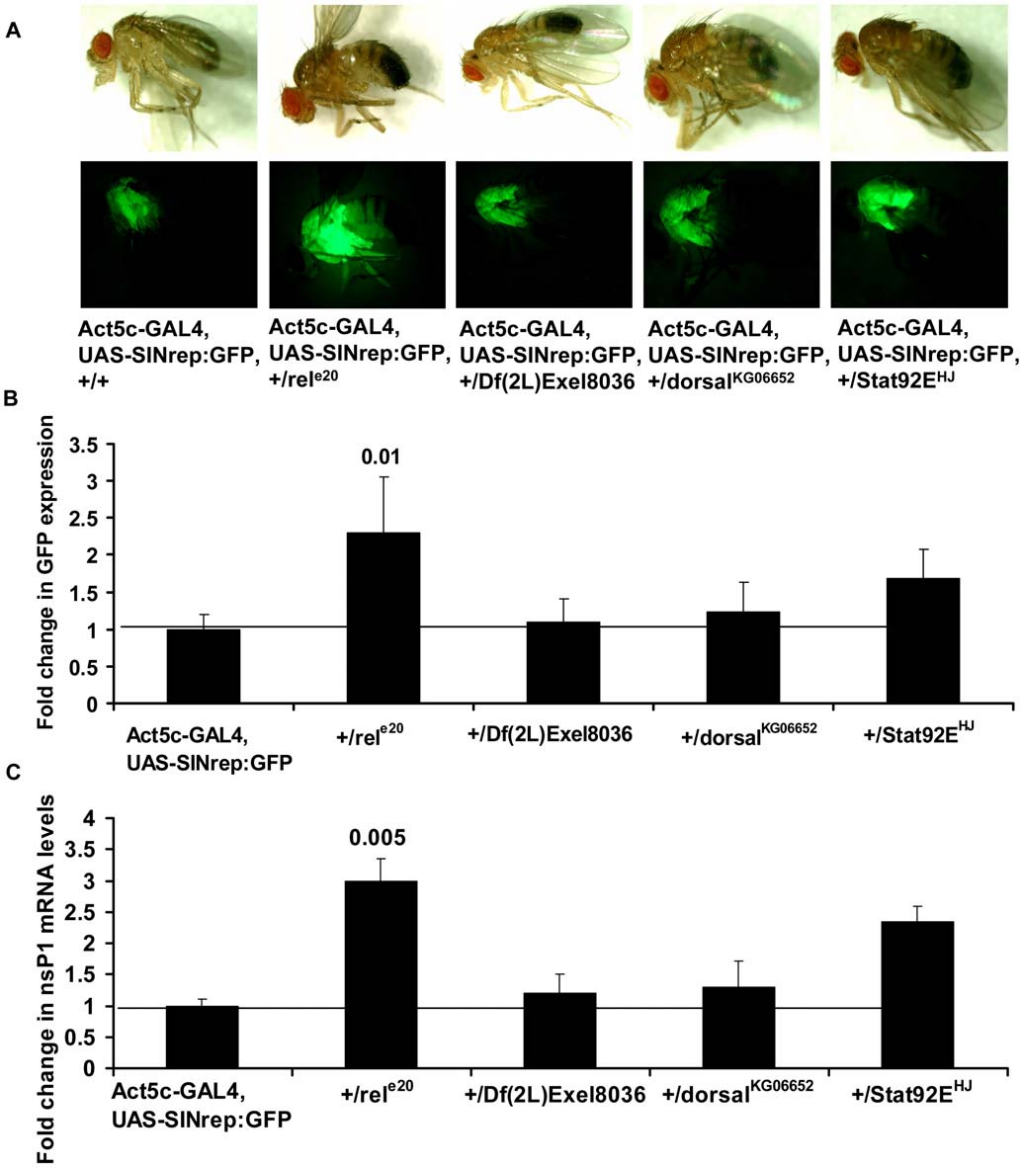
Alphaviral replication is higher in Relish mutant flies. (A) Bright field and fluorescence images of wild type SIN replicon flies and flies heterozygous for SIN replicon and *relish* (*rel^e20^*), *dif* (Df(2L)Exel8036) *dorsal* (*dorsal^KG06652^*), and *stat* (s*tat92E^HJ^*) mutations. (B) GFP expression levels in the above flies measured by fluorometry. The value obtained for control SIN replicon flies was considered as one. Data shown is representative of three independent experiments. (C) SIN replication was measured by real-time qRT-PCR analysis of nsP1 mRNA in control SIN replicon flies and flies heterozygous for SIN replicon and *relish* (*rel^e20^*), *dif* (Df(2L)Exel8036) *dorsal* (*dorsal^KG06652^*), and *stat* (s*tat92E^HJ^*) mutations. The value obtained for control SIN replicon flies was considered as one. Error bars represent SD.

The antimicrobial pathways in *Drosophila* play a very important role in combating infections. The Toll pathway results in the activation of NF-κB homologues Dif and Dorsal, the Imd pathway activates Relish, while Jak-STAT pathway triggers STAT. These transcription factors are central to the pathways, in the sense that they activate the antimicrobial effector molecules that eventually eradicate microbes. To determine if any of the known antimicrobial transactivators function to inhibit SIN RNA replication we crossed the SIN replicon fly with Dif, Dorsal, Relish and STAT mutants and examined their effects on SIN RNA replication. We measured GFP expression in F1 progeny as a gauge of viral RNA replication. In flies heterozygous for SIN replicon and *dif* or *dorsal* mutations there was no change in the levels of GFP. However, there was a 2.3-fold increase in GFP levels in SIN replicon flies heterozygous for a *relish* mutation (Figure 4A and 4B). SIN replication measured by qRT-PCR of nsP1 mRNA showed similar results. Levels of nsP1 mRNA was 3- fold higher in flies heterozygous for relish mutation compared to wt fly background (Figure 4C). This result suggests that Relish-dependent transcription may be involved in the suppression of SIN replication. Flies heterozygous for SIN replicon and *stat* also displayed increased SIN replication. GFP expression levels were 1.7- fold higher in STAT mutant flies compared to SIN replicon flies, suggesting that STAT might also play a role in inhibiting SIN RNA replication.

### Imd pathway is involved in defense against alphavirus

**Figure 5:**
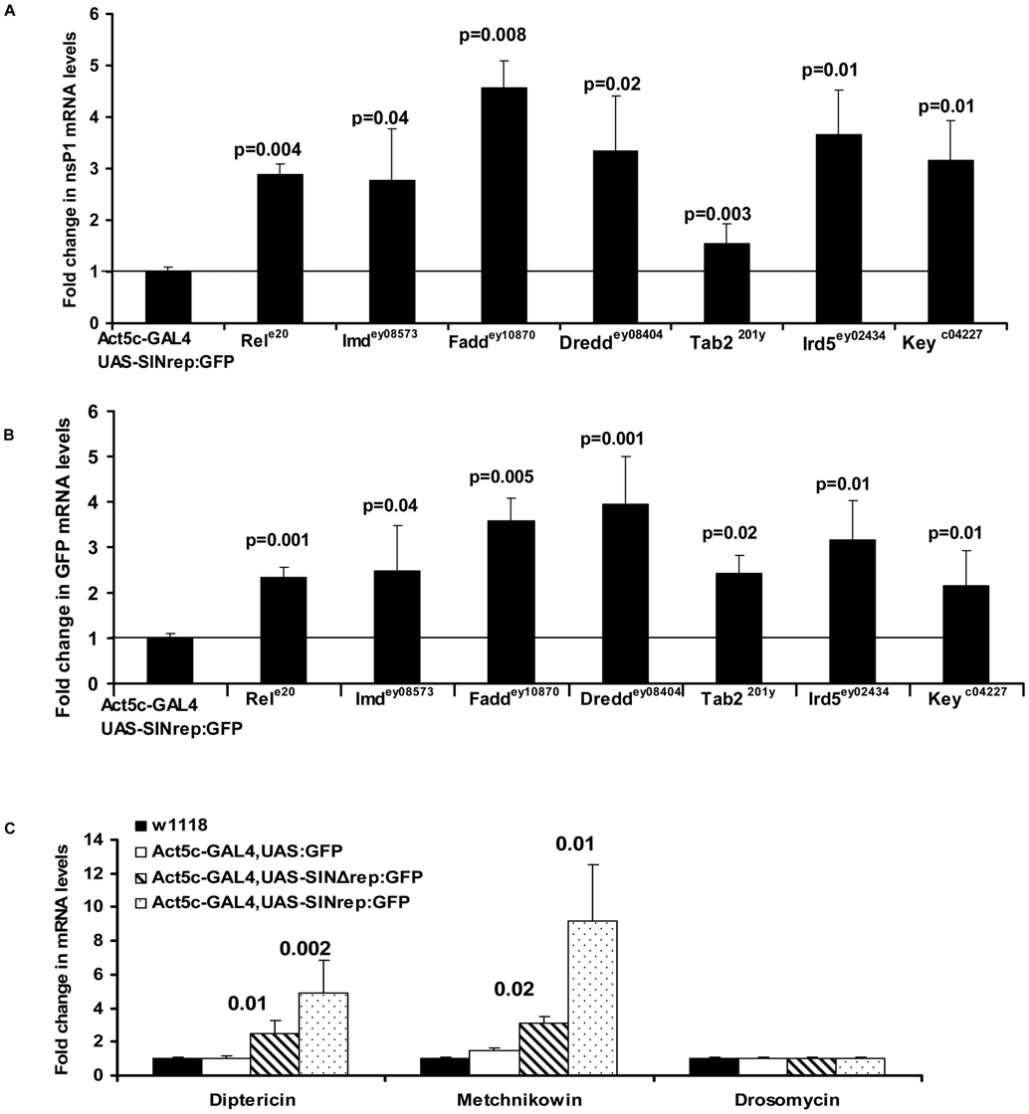
The Imd pathway is involved in controlling alphaviral replication. SIN replication was measured by real-time qRT-PCR analysis of nsP1 (A) and GFP (B) mRNAs in control SIN replicon flies and flies heterozygous for SIN replicon and *relish* (*rel^e20^*), *imd* (*imd ^ey08573^*), *dfadd* (*BG4^EY10870^*), *dredd* (*dredd^EY08404^*), *tab2* (*tab2 ^201y^*), *ird5* (*ird5^EY02434^*) and kenny (*key^c02831^*) mutations. The value obtained for control SIN replicon flies was considered as one. (C) The expression of Diptericin, Metchnikowin and Drosomycin AMPs was measured in w^1118^ flies, GFP expressing flies, mutant SIN replicon expressing flies, and SIN replicon expressing flies by real-time qRT-PCR. The value obtained for w^1118^ flies was considered as one. Data shown is representative of three independent experiments. Error bars represent SD.

Relish is activated as a result of signaling through the Imd pathway, therefore the data above indicated that this pathway is involved in an antiviral response. To determine the role of the Imd pathway in the suppression of SIN RNA replication we crossed the SIN replicon fly to flies mutant in upstream components of the Imd pathway and examined their effect on virus replication. SIN replication was measured by qRT-PCR of nsP1 mRNA. Replication of SIN RNA increased in flies containing mutations in the Imd pathway (Figure 5A). The levels of nsP1 transcript were 2.8, 2.7, 4.5 and 3.3- fold higher in F1 progeny heterozygous for *relish*, *imd*, *dfadd* and *dredd* respectively when compared to the replication in a wt fly background. Similarly, nsP1 mRNA levels were also higher by 1.5, 3.6 and 3.1- folds in flies heterozygous for *tab2*, *ird5* and *key* respectively compared to levels in wt flies. We also measured SIN replication via levels of GFP transcript. The levels of GFP transcripts were 2.3, 2.4, 3.7 and 3.9- fold higher in F1 progeny heterozygous for *relish*, *imd*, *dfadd* and *dredd* respectively when compared to the replication in a wt fly background. GFP mRNA levels were also higher by 2.4, 3.1 and 2.1- folds in flies heterozygous for *tab2*, *ird5* and *key* respectively compared to levels in wt flies (Figure 5B). These data demonstrate that the Imd pathway plays a role in the control of SIN genome replication.

The activation of Relish leads to transcription of the AMPs Diptericin and Metchnikowin, while activation of Toll pathway leads to the expression of Drosomycin. Levels of these transcripts were used as markers of antimicrobial pathway activation. A large induction of both Diptericin and Metchnikowin was detected in SIN replicon flies. Diptericin was up by 4.8- fold and Metchnikowin by 9.2- fold as compared to w^1118^ flies. However, there was no difference in the levels of Drosomycin in SIN replicon flies when compared to w^1118^ flies. These results confirmed that SIN replicon was stimulating the Imd pathway that activated Relish and expression of AMPs. We also examined the expression of these AMPs in mutant SIN replicon flies expecting no increase in Relish-dependent mRNA expression of AMPs in these flies since viral replicon replication was not occurring. However, a 2.4 and 3.1- fold increase in *diptericin* and *metchnikowin* transcripts respectively was detected in the mutant SIN replicon flies (Figure 5C). This suggests that viral replication is being detected through recognition of the viral RNA and replication is not necessary for stimulation of this pathway. It is important to note that significant increases in the levels of AMP encoding mRNAs were not observed as a consequence of UAS/GAL4 based GFP expression, demonstrating that Relish activation is not occurring simply as a consequence of over-expression of a heterologous gene (Figure 5C).

### Relish mutant flies have higher viral loads

**Figure 6:**
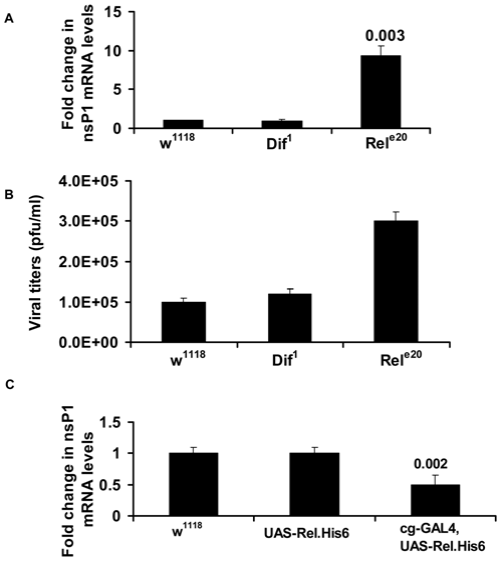
Alphaviral replication in Relish flies. (A) SIN virus replication was measured by real-time qRT-PCR analysis of nsP1 mRNA in control w^1118^ flies, *dif* (*dif^1^*) and *relish* (*rel^e20^*) mutant flies. (B) Viral titers of SIN in w^1118^, Dif and Relish mutant flies 5 days post infection. (C) SIN virus replication in control w^1118^, UAS-Relish.his6 flies and flies over-expressing Relish (cg-GAL4,UAS-Relish.His6). Replication was measured by qRT-PCR analysis of nsP1 mRNA. Flies were infected with 200pfu of SIN:GFP virus. Viral replication and viral titers were measured five days post infection. Data shown is representative of three independent experiments. Error bars represent SD.

Finally to confirm the role of Relish in antiviral defense against alphavirus in *Drosophila*, we infected *relish^−/−^* flies, *dif^−/−^* intrathoracically with 200 pfu of SIN. Viral loads were measured by levels of RNA containing nsP1 sequence. Relish mutant flies had 9.3- fold higher level of viral RNA compared to w^1118^ flies 5 days post-infection (Figure 6A). The levels of viral RNA however remained the same in *dif^−/−^* flies as compared to w^1118^ flies confirming that Imd but not Toll pathway is involved in controlling SIN infection. Also, SIN viral titers were 3-fold higher in *relish^−/−^* flies compared to w^1118^ and *dif^−/−^* flies (Figure 6B).

The anti-viral role of relish was also verified by overexpressing relish. UAS-Relish.his6 flies were crossed to a hemocyte GAL4 driver. The hemocyte driver was chosen to maximize expression of Relish [43]. The F-1 progeny overexpressing Relish were injected with 200 pfu of SIN virus intrathoracically and viral replication was measured five days post- infection. The levels of viral RNA in flies overexpressing Relish was down by 0.51-fold as compared to wt w^1118^ flies or UAS-Relish.his6 flies without GAL4 driver (Figure 6C).

### SIN activates Relish in mosquito cells

**Figure 7:**
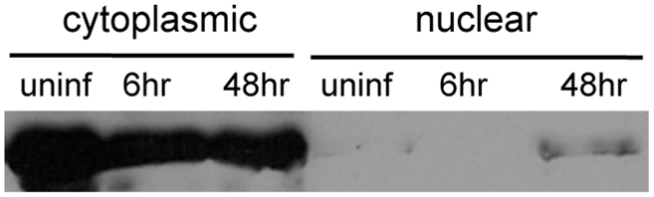
Relish activation in mosquito cells. Western blot with anti-Relish antibody on cytoplasmic and nuclear extracts of c6/36 cells infected with SIN. C6/36 cells were not infected or infected with SIN for 6 and 48 h and lystes obtained and fractionated and probed for Relish.

The *Drosophila* Relish consists of an N-terminal Rel/NF-κB homology domain (RHD) and a C-terminal IκB-like domain with ankyrin repeats. Relish is activated by endoproteolytic cleavage, the RHD translocates to the nucleus and the IκB domain is retained in the cytoplasm. The RHD binds to DNA and activates the transcription of AMPs [44]. Although the exact mechanism of activation of mosquito Relish is still not known, mosquitoes produce three isoforms of Relish from the *rel2* gene by differential mRNA splicing. The first Relish isoform resembles the *Drosophila* Relish; it contains the RHD and IκB -like domain. The second isoform has a RHD but lacks the IκB-like domain but has a unique 3′-UTR. The third isoform lacks the RHD but has an intact IκB-like domain. To verify the relevance of our findings in *Drosophila* we examined the cellular localization of the N-terminal RHD in uninfected and SIN infected cultured mosquito cells (c6/36). Infected and uninfected cells were fractionated into cytoplasmic and nuclear fractions and quantities of the RHD were examined by western blot. While levels of the RHD were consistently high in the cytoplasm of both infected and uninfected cells, we observed that SIN infection repeatedly resulted in an increase in the amount of the RHD in the nucleus of infected cells following 48 h of infection (Figure 7). This strongly implies that SIN activates Relish-mediated transcription during persistent infection of cultured mosquito cells.

## Discussion

In the present study we have developed a powerful system to genetically examine the effects of host factors in suppressing SIN RNA replication in *Drosophila*. SIN RNA replication was launched by cellular transcription of the DNA copy of the viral genome using the UAS-GAL4 system. We crossed the SIN replicon fly to flies carrying mutations in specific components of antimicrobial pathways to determine their role in anti-SIN defense. Replication of SIN RNA was higher in flies heterozygous for a mutation in *relish* (Imd pathway) but not for *dif* or *dorsal* (Toll pathway). Additionally, SIN replication was higher in flies heterozygous for upstream components of the Imd pathway. Furthermore, intrathoracic injections of SIN virus into *relish^−/−^* flies showed higher viral loads and enhanced replication whereas SIN replication was unchanged in *dif^−/−^* flies. These findings indicate that the Imd pathway is involved in antiviral defense against SIN. This is the first report of the Imd pathway's involvement in antiviral defense in *Drosophila*.

The data presented in this manuscript demonstrated that SIN replicon mediated RNA synthesis could be launched from the *Drosophila* genome using the UAS-GAL4 system. Using the Act5C-GAL4 driver we observed robust SIN RNA replication at numerous stages of development. The replicon replication was not pathogenic, although there was 1–2 day delay in the developmental cycle. The pattern of GFP expression resulting from the SIN replicon was different from that observed in the control UAS-GFP fly and was not ultimately defined by the pattern of driver expression. We hypothesize that cells that contained replicating replicon RNA at an early stage in development, when act5C expression is ubiquitous [42], continued to host viral RNA synthesis at later developmental stages even in the absence of primary transcription of the replicon RNA from the fly genome. The pattern of GFP expression in the SIN replicon containing flies also showed that not all tissues are permissive for virus genome replication. For instance while Act5C-GAL4 drives primary UAS-dependent transcription in the abdomen, the lack of GFP signal in the abdomen of replicon-containing flies suggests tissues in this body segment are significantly less permissive for virus replication than thoracic muscle in which replicon derived GFP expression was high.

This system for the launch of SIN RNA replication allows a significant amount of control over where and when replicon RNA is produced in the developing fly. By using different GAL4 drivers we can launch replicon RNA production in a temporal and spatially specific fashion. This provides a greater degree of control than with other transgenic systems of virus launch [24],[40] in which a generalized heat-shock is used to induce cell-mediated transcription of the viral RNA. This flexibility has allowed us to begin to map the permissivity of tissues for viral RNA replication during fly development, including salivary glands, muscle, mid-gut, and CNS (data not shown). A consistent finding when analyzing viral RNA replication using different drivers to launch SIN replicon replication has been that thoracic muscle is highly permissive for virus replication confirming our initial findings with the Act5C driver.

Additionally this system allows us to genetically screen for host factors that are both pro- and antiviral. Our confirmation of the previously reported involvement of the RNAi pathway in the control of SIN replication led us to examine the role other antimicrobial pathways play in the control of SIN RNA synthesis [24]. Examination of the effects of transcription factors associated with antimicrobial signaling pathways revealed SIN replicon replication was 2.3- fold higher in flies heterozygous for Relish mutation. Relish is the terminal transcription factor in the Imd signaling cascade. This pathway is usually activated by Gram-negative bacteria, however activation can also occur by some fungi that do not activate the Toll pathway [45]. Our observation of enhanced RNA replication in flies deficient in components of the Imd pathway, in combination with the observed increase in Relish dependent transcription in flies harboring SIN replicon RNA, has for the first time demonstrated that Imd/Relish pathway is activated by a virus. Earlier studies have indirectly implied a role for viruses in activation of the Imd pathway but have not found specific antiviral effects. Zambon et.al. found that DXV activates sets of AMPs transcribed by both Relish and Dif but only the Toll pathway was involved in anti-DXV defense [33]. Sanders et.al. studied the transcriptional expression profiles of mosquito midguts infected with SIN. Based on the expression pattern they hypothesized that early innate immune responses to SIN infection was through Toll pathway, which is later shut-off and the Imd pathway is activated later in infection [38]. Also, RNAi mediated silencing of native regulators- Cactus (Toll) and Casper (Relish) in mosquitoes followed by infection with Dengue virus activated a considerable number of genes by Relish, however only genes activated by Dif had anti-Dengue effect [39]. Our analyses of Dif and Dorsal imply that the Toll pathway does not have an antiviral effect at the level SIN RNA replication.

While we have strong evidence that the Imd pathway is activated in response to SIN RNA, how the Imd pathway is activated by SIN remains an open question. Gram-negative bacteria activate the Imd pathway when bacterial proteoglycans are recognized by host transmembrane receptors - PGRP-LC and PGRP-LE. This binding leads to the recruitment of Imd by an unknown protein [16]. The SIN replicon does not produce proteins resembling the peptidoglycans of bacteria. We therefore assumed that PGRPs have no role in SIN replication. To address this assumption we measured SIN replication in PGRP-LE and LC mutant flies and we found no difference in the replication of SIN in flies heterozygous for PGRP-LE and LC (Figure S2). We believe that in the SIN replicon flies, the activation of the Imd pathway occurs within the cell since the replication complexes and the replicating RNA are intracellular. We therefore hypothesized that an intracellular receptor recognizes either viral RNA or replication complexes and feeds a signal into the Imd pathway that ultimately activates Relish. Dicer-2 serves as a cytoplasmic sensor of viral RNA similar to mammalian RIG-I and Mda5 and induces the expression of antiviral protein Vago [46]. Since our data indicate that viral RNA is responsible for Relish activation, we examined the role of Dicer-2, and also Dicer-1 in activation of the Imd pathway. However to this point, we have observed no role for Dicer-2 or Dicer-1 in induction of Relish-mediated transcription in SIN replicon flies (Figure S3).

It is currently unclear what the effectors of the Relish-mediated anti-SIN response might be. While increased transcription of the AMP genes *diptericin* and *metchnikowin* was used as a measure of Relish activation, a role for these AMPs in antiviral immunity seems unlikely. The induction of AMP expression as a consequence of SIN replication may however be an important prophylactic immune response preventing secondary bacterial infection due to virus-induced tissue damage. We are currently performing comparative transcriptome analyses to identify differences in transcript levels unique to the SIN replicon flies in order to facilitate the identification of antiviral effector molecules.

Another variable that may significantly affect the antiviral response of *Drosophila* is the presence of *Wolbachia*. *Wolbachia* are Gram-negative bacteria that manifest intracellular, inheritable infections. In *Drosophila melanogaster* the infection is transmitted vertically through the female, and previous studies have reported that *Drosophila* infected with *Wolbachia* are less susceptible to infections with RNA viruses [47],[48]. We tested all the lines used in this study for presence of *Wolbachia* by PCR. Among the lines tested two lines were positive for *Wolbachia*, the Dif mutant line (w[1118];Df(2L)Exel8036/CyO) and PGRP-LC mutant (w^67c23^ P{lacW}l(1)G0414^G0414^/FM7c) line (Figure S4). In our crosses we used females from the SIN replicon expressing line (Act5C-GAL4/UAS-SINrep:GFP) that were negative for *Wolbachia* and males from the mutant lines. Since *Wolbachia* is transmitted maternally, the progeny resulting from these crosses are not infected with *Wolbachia*, and hence virus replication was consistently analyzed in a *Wolbachia* negative background.

SIN replication was enhanced in STAT mutant flies suggesting that the Jak/STAT pathway may also be involved in controlling SIN replication. Previous data have shown DCV infection induces the expression of *vir-1* and expression of *vir-1* is dependent on Hopscotch- the Jak kinase. Further genetic experiments suggested that Hopscotch was required but not sufficient for the induction of DCV -regulated genes [31]. It is possible that SIN infection activates both Imd and Jak/STAT pathways and that multiple pathways are required for effective viral clearance. However the potential role of Jak/STAT in SIN infections needs to be completely understood.

While *Drosophila* represents a genetically accessible model organism, alphaviruses are naturally transmitted between vertebrate hosts by mosquitoes. The mosquito genome has orthologous genes for *dif* and *relish*; *rel1* and *rel2* respectively [49]–[52]. We verified the relevance of our findings in *Drosophila* by infection of cultured mosquito cells. The results indicated that Rel-2 is activated during SIN infection of c6/36 cells. The RHD containing isoforms of Rel-2 localize to the nucleus later during infection. These results imply that Relish-mediated transcription may be important in controlling virus replication during the persistent phase of infection in mosquitoes. These results also suggests that the results generated by using *Drosophila* as model organism can be compared and verified in mosquito cells.

In summary, we have developed a system for the controlled launch of SIN RNA replication from the genome of *Drosophila*. Using this system we have demonstrated that, in addition to RNAi, Jak/STAT, and Toll, the Imd pathway plays an important role in the antiviral response in flies. Further characterization of how the virus is recognized by the host and what downstream effector molecules are required for the control of virus replication will provide additional insights into the role of this pathway in particular and the antiviral response of arthropods in general.

## Materials and Methods

### Cells and virus

BHK-21 and C6/36 cells (American Type Culture Collection) were grown in Alpha MEM and L15 media respectively (Invitrogen) supplemented with vitamins 10% of fetal bovine serum or heat inactivated FBS (C6/36). SIN:GFP is wild type SIN expressing GFP from a second subgenomic promoter was generated by transfection of BHK-21 cells with in-vitro transcribed infectious SIN:GFP TE RNA [53].

### Plasmid construction and transgenic flies

The pUAST- SINrep: GFP plasmid was constructed by replacing the Sbf1 and Not1 fragment of pUAST vector with pSINrep/GFP that encodes the non-structural proteins and GFP from a sub-genomic promoter preceded by 5 UAS sequences. The pUAST- SINΔrep: GFP construct was made by deleting 5.7 kb fragment in the non-structural region of pUAST- SINrep: GFP. RsrII and KpnI were used to remove the 5.7 kb fragment, and the remaining product gel purified and treated with DNA polymerase I large (Klenow) fragment (NEB) to remove the 3′ overhang and fill-in the 5′ overhang. The plasmid was the phenol/chloroform extracted, precipitated and ligated using T4 DNA ligase (NEB). Stable transgenic lines harboring the UAS-SIN constructs were generated via standard methods [54]. We obtained a transformant line for SINrep: GFP that mapped to the third chromosome and one line for SINΔrep: GFP that mapped to the second chromosome.

### Fly strains

Fly lines (listed in Table S1) were obtained from the Bloomington stock center. *dcr-1^Q1147X^* and *dcr-2^L811FXS^* flies were provided by R Carthew (Northwestern University). *Dif^1^* were provided by D Ferrandon (Institut de Biologie Moléculaire et Cellulaire). Fly stocks were raised on standard cornmeal–agar medium at 25°C.

### Microscopy and imaging

Live flies, pupae and larvae were anesthetized with CO_2_ and viewed under on a Nikon SMZ1500 dissecting microscope. Photographs were taken using Nikon DXM1200 camera.

### GFP quantitation

Five adult flies were homogenized in 10 mM Tris (pH 8.4), 100 mM NaCl, 1 mM MgCl2, 10 mM dithiothreitol [55]. Homogenates were centrifuged at 15000 g for 5 min to remove debris and fluorescence was detected using a Synergy 4 HT Multi-Detection Microplate Reader (Biotek) with excitation filter set to 485 nm and emission filter at 520 nm.

### Viral injections

For viral injections, flies were anesthetized with CO_2_ and injected with SIN:GFP virus or control alpha MEM media in the thorax using a glass capillary needle. To estimate the number of viral plaque forming units injected into flies, injected flies were immediately flash frozen in liquid nitrogen, homogenized in PBS, centrifuged at 15000 g for 15 minutes to remove the debris and viral titers determined by plaques assays of homogenates. Approximately 200 pfu of Sin:GFP virus was injected into the flies. Five days post-infection flies were collected and viral titers determined as mentioned above. For the survival experiments, the injected flies were put on fresh food, and the number of surviving flies was counted at regular intervals.

### Real time quantitative RT-PCR analysis

RNA was extracted by homogenizing flies in TRIzol reagent (Invitrogen). cDNA was made using AffinityScript QPCR cDNA synthesis kit (Stratagene), and PCR amplification was done using Brilliant II SYBR Green QPCR master mix (Stratagene) following manufacturer's protocol. Gene expression was normalized to the actin mRNA expression. The comparative threshold cycle (C_T_) method was used to determine fold changes of transcript present in samples. Oligonucleotides used are listed in Protocol S1.

### Western blot analysis

C6/36 cells were not infected or infected with SIN:GFP virus at MOI of 0.1 for 6 h and 48 h. Western blot analysis was performed using standard procedures. Rabbit anti-N Rel antibody (kindly gifted by S. Stoven of Umea University) was used to detect Relish.

### Accession numbers

The FlyBase (http://flybase.org/) accession numbers for the genes used in the text include *actin5C* (CG4027), *dfadd* (CG12297), *dicer1* (CG4792), *dicer2* (CG6493), *dif* (CG6794), *diptericin* (CG12763), *dorsal* (CG6667), *dredd* (CG7486), *drosomycin* (CG10810), *imd* (CG5576), *ird5* (CG4201), *kenny* (CG16910), *metchnikowin* (CG8175), *pgrp-lc* (CG4432), *pgrp-le* (CG8995), *relish* (CG11992), *stat92E* (CG4257), *tab2* (CG7417).

## Supporting Information

Table S1 Fly Stocks From Bloomington Stock Center
(0.04 MB DOC)

Protocol S1 Supporting Materials and Methods
(0.03 MB DOC)

Figure S1 Minus-strand intermediates are made during SIN replication in SIN replicon flies. The production of minus strand intermediates during replication of SIN in SIN replicon (Act5C-GAL4,UAS-SIN:GFP) flies was measured by RT-PCR of nsP1. RNA from w^1118^ flies and BHK cells infected with SIN virus was used as negative and positive control respectively.
(0.91 MB TIF)

Figure S2 Alphaviral replication is not affected in PGRP LE or LC mutant flies. SIN virus replication was measured by real-time qRT-PCR analysis of nsP1 mRNA in SIN replicon flies and flies heterozygous for SIN replicon and PGRP-LC and LE. The value obtained for control SIN replicon flies was considered as one. Data shown is representative of three independent experiments. Error bars represent SD.
(0.26 MB TIF)

Figure S3 Dicer 1 or Dicer 2 do not activate IMD pathway through recognition of viral RNA. The expression of Diptericin and Metchnikowin AMPs was measured in flies heterozygous for SIN replicon and dicer 1 mutation (dcr-1^Q1147X^) or dicer 2 mutation (dcr-2^L811FXS^) by real-time qRT-PCR. The value obtained for SIN replicon flies was considered as one. Data shown is representative of three independent experiments. Error bars represent SD.
(0.65 MB TIF)

Figure S4 Detection of *Wolbachia* by PCR. The fly stocks used in the study were screened for presence of *Wolbachia* using PCR. The presence of *Wolbachia* was determined by PCR amplification of *wsp* gene.
(1.58 MB TIF)
