# Supplementary material for: A Novel System for the Launch of Alphavirus RNA Synthesis Reveals a Role for the Imd Pathway in Arthropod Antiviral Response
Source: PLoS Pathog. 2009 Sep 18;5(9):e1000582. doi: 10.1371/journal.ppat.1000582 (PMC2738967; doi:10.1371/journal.ppat.1000582)
Supplement: Table S1 — Fly Stocks From Bloomington Stock Center (0.04 MB DOC) [file ppat.1000582.s001.doc]

**Table S1**

| Stock number | Gene | Genotype |
| --- | --- | --- |
| 3954 | *act5c* | y^1^ w^*^; P{Act5C-GAL4}17bFO1/TM6B, Tb^1^ |
| 4440 | *tab2* | w^1118^; P{GawB}Tab2^201Y^ |
| 7835 | *dif* | w[1118]; Df(2L)Exel8036/CyO |
| 9457 | *relish* | w^1118^; Rel^E20^ e^s^ |
| 10456 | *dredd* | w^1118^;P{EP}Dredd^EP1412^ |
| 11044 | *kenny* | w^1118^; PBac{PB}key^c02831^ |
| 12014 | *pgrp-le* | w^67c23^ P{lacW}l(1)G0414^G0414^/FM7c |
| 12500 | *pgrp-lc* | w^1118^; P{GT1}PGRP-LC^BG00650^ |
| 14609 | *dorsal* | w^1118^; P{SUPor-P}dl^KG06652^/CyO, P{sevRas1.V12}FK1 |
| 17474 | *imd* | y^1^ w^67c23^; P{EPgy2}imd^EY08573^ |
| 19825 | *Ird5* | y^1^ w^67c23^; P{EPgy2}ird5^EY02434^ |
| 20638 | *dfadd* | y^1^ w^67c23^; P{EPgy2}BG4^EY10870^ pit^EY10870^ |
| 24510 | *stat92* | y^1^ w^*^; ry^*^ e^1^ Stat92E^HJ^/TM3, Sb^1^ |
