# Supplementary material for: A Novel System for the Launch of Alphavirus RNA Synthesis Reveals a Role for the Imd Pathway in Arthropod Antiviral Response
Source: PLoS Pathog. 2009 Sep 18;5(9):e1000582. doi: 10.1371/journal.ppat.1000582 (PMC2738967; doi:10.1371/journal.ppat.1000582)
Supplement: Protocol S1 — Supporting Materials and Methods (0.03 MB DOC) [file ppat.1000582.s002.doc]

**Supporting Materials and Methods:**

**Oligonucleotide primers used for real time quantitative RT-PCR**:

nsP1 forward, 5' -GGTTACACACAATAGCGAGGGCTT, and reverse, 5'-TGGTGTTCCTGTTAGTCCTACCGT-3',

GFP forward, 5'-AAGCTGACCCTGAAGTTCATCTGC-3', and reverse, 5'-CTTGTAGTTGCCGTCGTCCTTGAA-3',

Actin forward, 5'-ATGTGTGACGAAGAAGCATCAGCC-3', and reverse, 5'- TCATCCCAGTTGGTGATAATGCCG -3',

Metchnikowin forward, 5'- ATGCAACTTAATCTTGGAGCGA-3', and reverse, 5'-TGTGTTAACGACATCAGCAGTGTG-3',

Diptericin forward, 5'- AAGTGGGAAGCACCTACACCTACA-3', and reverse, 5'-GTTCCGGGTTAAACAAACAACGCC-3',

Drosomycin forward, 5'- CATTTACCAAGCTCCGTGAGAACC-3', and reverse, 5'- GATTTAGCATCCTTCGCACCAGCA-3'.

**Minus strand RT-PCR:** RNA was extracted by homogenizing flies or BHK cells in TRIzol reagent (Invitrogen). Minus strand cDNA was synthesized using 5’ nsP1 forward primer (5’-ATGGAGAAGCCAGTAGTAAACGTAGACG-3’) that specifically anneals to SIN minus-strand RNA. PCR amplification of nsP1 was performed using the above 5’ nsP1 forward primer and 3’ nsP1 reverse primer (5’TCATTATGCTCCGATGTCCGCCT GG-3’).

***Wolbachia* PCR**: Genomic DNA was extracted by homogenizing flies in 25 mM NaCl, 10 mM Tris-Cl pH=8.0, 1 mM EDTA, 200 μg/ml proteinase K and incubated for 30 min at 37 °C. Proteinase K was inactivated at 95 °C for 5 min. Lysates were spun and supernatant was used for PCR. *Wolbachia* in fly extracts was detected by primers specific to wolbachia surface protein (*wsp*) gene. wsp 81F (5′-TGGTCCAATAAGTGATGAAGAAAC-3′) and wsp 691R (5′-AAAAATTAAACGCTACTCCA-3′) [1].

**Supporting references:**

1. Zhou W, Rousset F, O'Neil S (1998) Phylogeny and PCR-based classification of Wolbachia strains using wsp gene sequences. Proc Biol Sci 265: 509-515.
