# Supplementary figures and images for: A Novel System for the Launch of Alphavirus RNA Synthesis Reveals a Role for the Imd Pathway in Arthropod Antiviral Response
Source: PLoS Pathog. 2009 Sep 18;5(9):e1000582. doi: 10.1371/journal.ppat.1000582 (PMC2738967; doi:10.1371/journal.ppat.1000582)

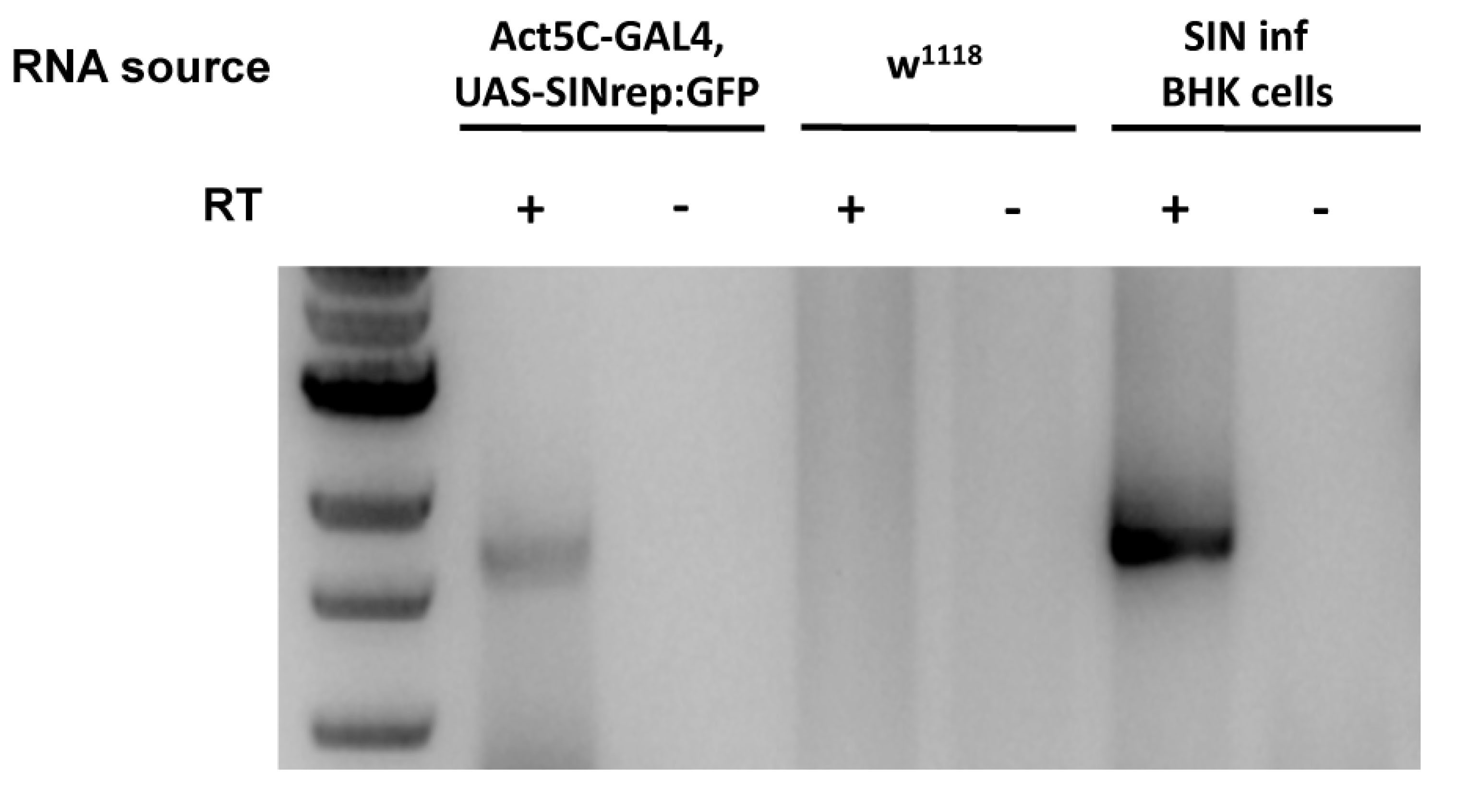

Supplement: Figure S1 — Minus-strand intermediates are made during SIN replication in SIN replicon flies. The production of minus strand intermediates during replication of SIN in SIN replicon (Act5C-GAL4,UAS-SIN:GFP) flies was measured by RT-PCR of nsP1. RNA from w1118 flies and BHK cells infected with SIN virus was used as negative and positive control respectively. (0.91 MB TIF) [file ppat.1000582.s003.tif]

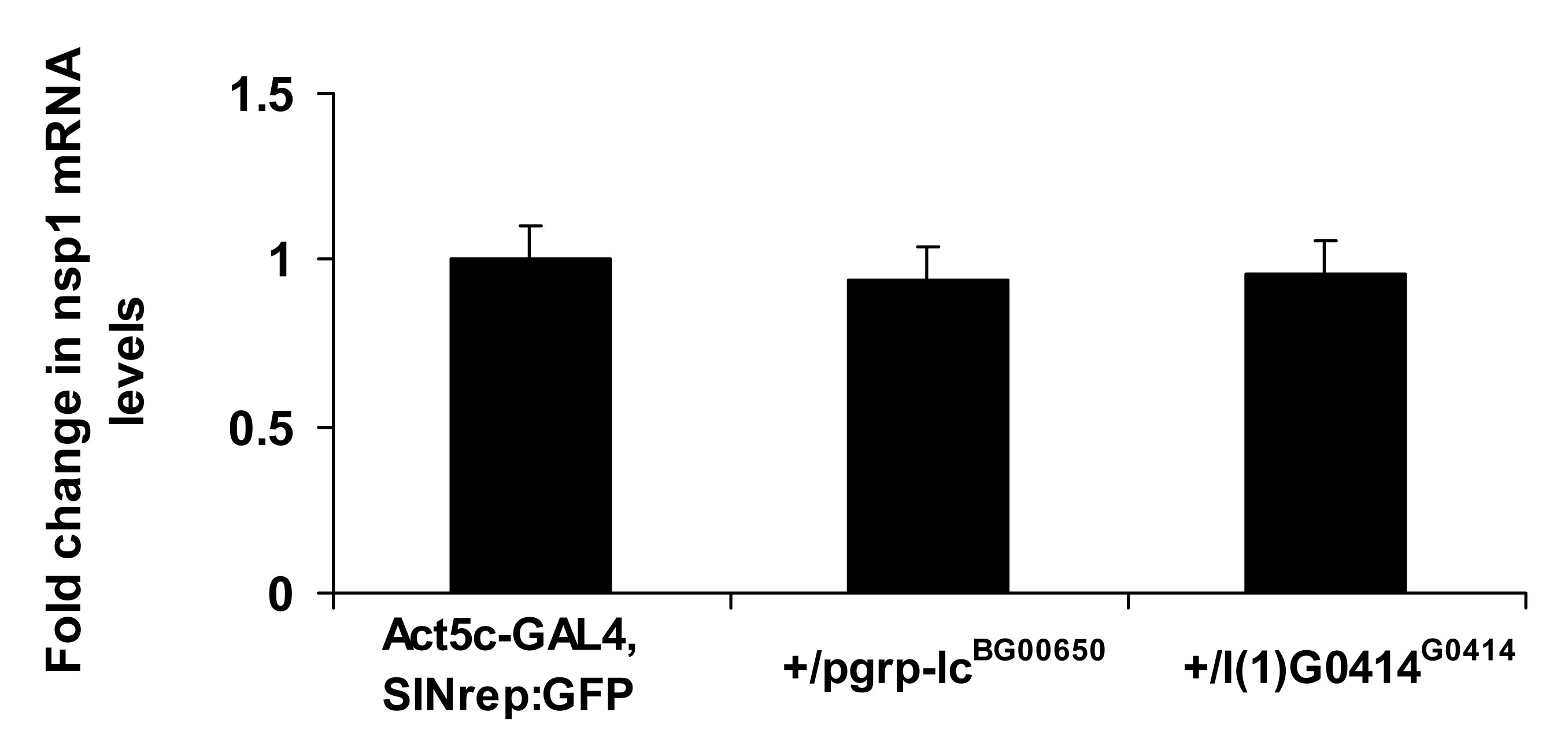

Supplement: Figure S2 — Alphaviral replication is not affected in PGRP LE or LC mutant flies. SIN virus replication was measured by real-time qRT-PCR analysis of nsP1 mRNA in SIN replicon flies and flies heterozygous for SIN replicon and PGRP-LC and LE. The value obtained for control SIN replicon flies was considered as one. Data shown is representative of three independent experiments. Error bars represent SD. (0.26 MB TIF) [file ppat.1000582.s004.tif]

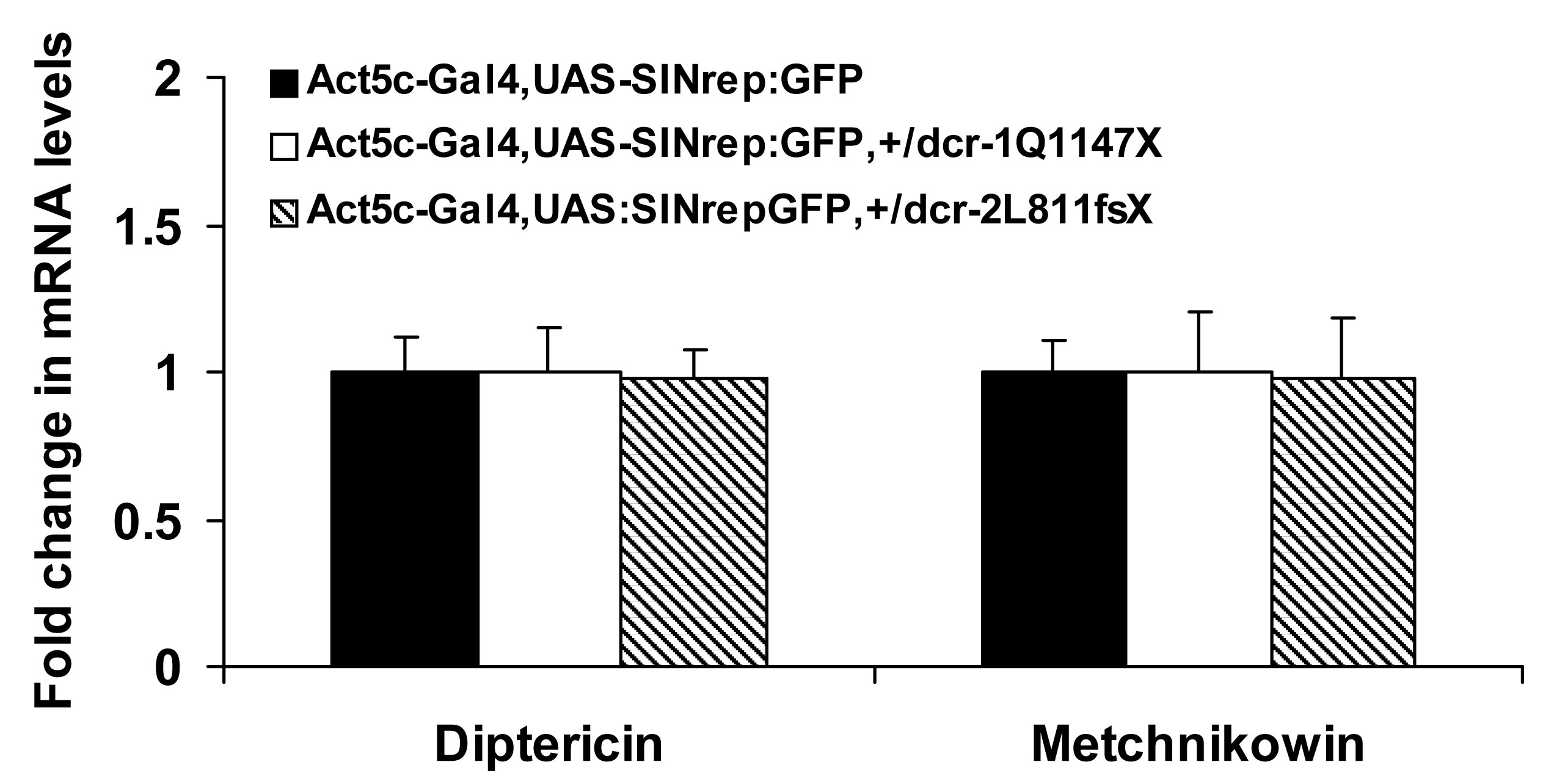

Supplement: Figure S3 — Dicer 1 or Dicer 2 do not activate IMD pathway through recognition of viral RNA. The expression of Diptericin and Metchnikowin AMPs was measured in flies heterozygous for SIN replicon and dicer 1 mutation (dcr-1Q1147X) or dicer 2 mutation (dcr-2L811FXS) by real-time qRT-PCR. The value obtained for SIN replicon flies was considered as one. Data shown is representative of three independent experiments. Error bars represent SD. (0.65 MB TIF) [file ppat.1000582.s005.tif]

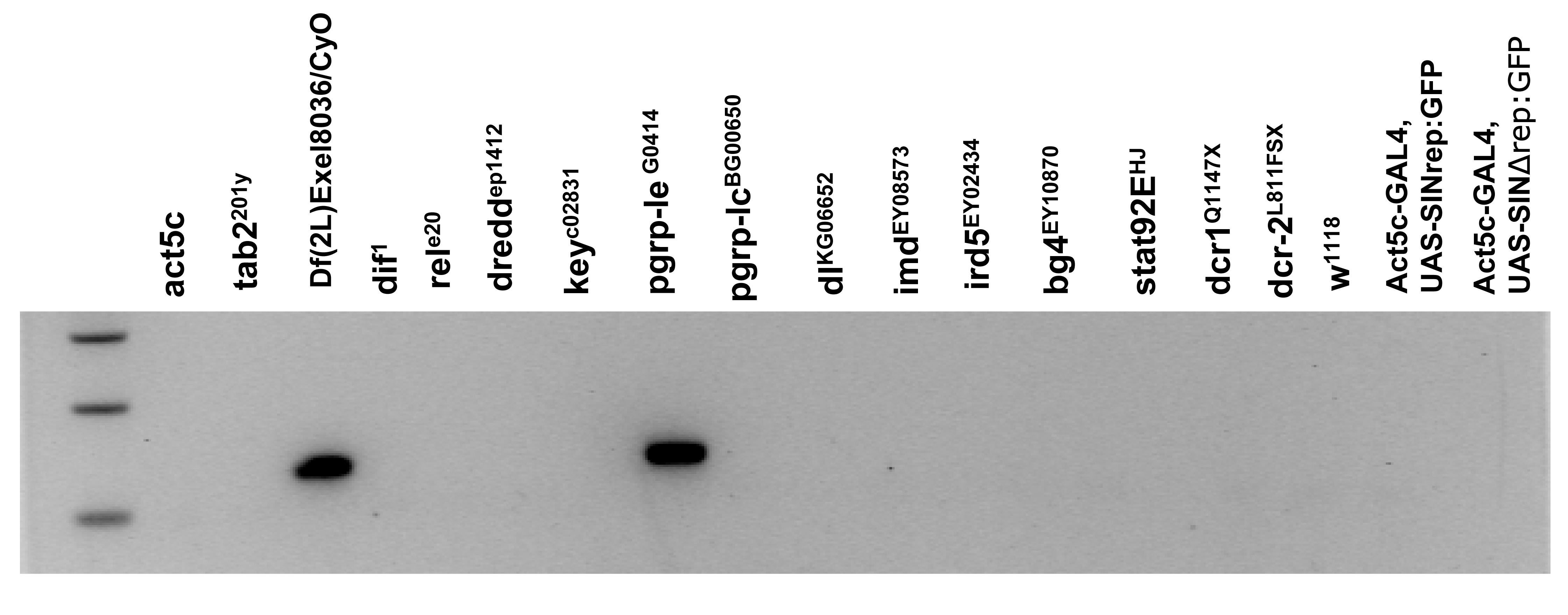

Supplement: Figure S4 — Detection of Wolbachia by PCR. The fly stocks used in the study were screened for presence of Wolbachia using PCR. The presence of Wolbachia was determined by PCR amplification of wsp gene. (1.58 MB TIF) [file ppat.1000582.s006.tif]
